# Some Remarks on Hernia

**Published:** 1908-10-10

**Authors:** 


### Surgery.

### SOME REMARKS ON HERNIA.

### II.—THE DIAGNOSIS AND TREATMENT OF INGUINAL HERNIA.

The diagnosis of an inguinal hernia is, ordinarily speaking, a matter of the greatest ease. The patient complains of the presence of an abnormal swelling in the inguinal region which has suddenly made its appearance either after a recognised strain or for no apparent reason. That is what troubles him most. There may or may not be concomitant symptoms, the commonest of which is a dragging pain in the hypogastrium, which is caused by the descended coils dragging on the attachment of the mesentery to the posterior abdominal wall.

On examination an oval swelling is found, whose apex is emerging for a variable distance from the inguinal canal. It may be so small that it only just comes through the external abdominal ring, or, on the other hand, it may descend to the bottom of the scrotum. This depends largely on the length of time for which it has persisted, since it tends gradually to increase in size in the course of time. The main diagnostic feature on which reliance is placed is the presence of an expansile impulse communicated to the hernia when the patient coughs; and it is useful to remember that this impulse will be more easily appreciable to a finger applied to the apex of the swelling than to the neck. This is a simple point, but one which is not always observed. A slight pressure will cause the swelling to return to the abdomen. This is known as reduction. It is said to be accompanied by a gurgle: as a matter of fact anything approaching a genuine gurgle is very rarely obtained, but the reduction of a coil of intestine from a hernial sac gives a sensation to the hand which is quite unlike anything else in surgery, and one which even a small experience enables one to appreciate.

That is the picture of a simple uncomplicated case, but an infinite number of variations from the type are found. Thus instead of intestine, the sac may contain omentum (this is known as an epiplocele). It feels soft and doughy to the examining hand, has a less distinct impulse, and returns to the abdomen gradually; or the sac may contain both intestine and omentum, the latter being adherent to the sac-wall. In this case, as will be readily understood, the hernia is only partially reducible.

Unless there are adhesions between the sac wall and its contents, an inguinal hernia tends to become reduced spontaneously when the patient lies down. Thus it is not uncommon for a patient to say that the swelling disappears during the night, but comes down again as soon as he begins to get about and pursue his avocation. If the patient is seen when the hernia is fully reduced, it may be difficult to make up one's mind whether a genuine hernia has ever existed. Even if the patient be made to stand up and cough, it may not come down again; sometimes, of course, one may be able to feel the edge of the sac if it is at all thickened. In the absence of all evidence, however, one is forced to judge by the condition of the walls of the inguinal canal. In a normal individual the index finger can be inserted through the external abdominal ring as far as the base of the nail, but if the patient coughs, it can be felt to be squeezed out. If the external abdominal ring will admit two fingers, or if one finger is not expelled as the result of the patient's coughing, it may be regarded as presumptive evidence of the existence of an inguinal hernia.

Even when there is an obvious scrotal swelling the diagnosis is not invariably an easy matter, especially in a child, in whom a scrotal hernia may resemble a hydrocele of the tunica vaginalis. In both there is an oval swelling, which is translucent, for in infants the intestine itself is translucent; in both there may be an impulse, for it often happens that the upper pole of a hydrocele insinuates itself into the inguinal canal, and thus the result of any increase of intra-abdominal pressure is communicated to it. But, it will be said, the hydrocele is irreducible; but so may the hernia be, if there are any adhesions between the sac-wall and its contents; so that the problem presented may be a most difficult one. The differential diagnosis between an inguinal hernia and the other conditions which may be mistaken for one, or are said in text-books to resemble one, is an easy matter, and need not be discussed.

In coming to a conclusion as to the correct line of treatment, a great many factors must be carefully considered; they are (1) the patient's age; (2) the state of his general health; (3) his occupation; (4) the condition of the walls of his inguinal canal; and (5) the possibility or otherwise of controlling the hernia with any form of truss. Put shortly, the question of treatment resolves itself into this:—Is the patient to spend the remainder of his life wearing a truss, or is he to have his defect repaired once and for all by an operation? Let us take in order, then, the things which must be considered. (*a*) His age: Age in itself is not a contra-indication to operation, especially if that operation is going to enable the patient to spend his declining years in comfort. The successful results of prostatectomy in recent years is sufficient proof of this. (*b*) The state of his general health: Any severe constitutional malady such as albuminuria or glycosuria should make the surgeon hesitate, and in this case it is better either to wait, or to advise the patient that he must reconcile himself to a truss. (*c*) His occupation: If this is laborious it is an argument in favour of having a radical cure performed, since only then will the patient be able to pursue it in comfort or in safety. (*d*) The state of his abdominal wall: The inguinal canal is occasionally so weak that it may appear that no operation can mechanically strengthen it. This weakness in young patients is often congenital, but in those of a more advanced age is a senile change. But it must be remembered, as explained in the last article, that the essential part of a radical cure is the ligature and removal of the sac, and not the mechanical restitution of the abdominal wall.

